# Clinical characteristics, survival analysis and influencing factors of distant metastasis in patients with acromelanomas: A retrospective study

**DOI:** 10.1097/MD.0000000000038230

**Published:** 2024-06-07

**Authors:** Yan Fang, Zhu Yongqian, Lu Yin, Min Li, Zhang Mei, Yang Jing, Wu Di

**Affiliations:** a Department of Dermatology, Nanjing Pukou People’s Hospital, Nanjing, Jiangsu, People’s Republic of China; b Department of Information, Jiangsu Provincial People’s Hospital, Nanjing, Jiangsu, People’s Republic of China; c Department of Ultrasound, Jiangdong Community Service Center, Nanjing, Jiangsu, People’s Republic of China; d Department of Pathology, Nanjing Pukou People’s Hospital, Nanjing, Jiangsu, People’s Republic of China; e Department of Dermatology, Jiangsu Provincial People’s Hospital, Nanjing, Jiangsu, People’s Republic of China.

**Keywords:** acral melanoma, COX regression model, distant metastasis, influencing factors, prognosis, survival

## Abstract

The prognosis of acromelanomas (AM) is worse. The objective of this study was to investigate the clinical features of distant metastasis of AM and the factors affecting the survival and prognosis of patients. In this study, a retrospective study was conducted to select 154 AM patients admitted to Nanjing Pukou People’s Hospital from January 2018 to April 2021 for clinical research. The clinical characteristics of distant metastasis were statistically analyzed, and the survival curve was drawn with 5-year follow-up outcomes. The median survival time of the patients was calculated, and the clinicopathological features and peripheral blood laboratory indexes of the surviving and dead patients were analyzed. Logistic regression model was used to analyze the risk factors affecting the prognosis of AM patients. In this study, 154 patients with AM were treated, including 88 males and 76 females, aged from 27 to 79 years old, with an average age of (59.3 ± 11.7) years old. Among them, 90 cases had distant metastasis. The main metastatic sites were lung (47.78%) and lymph nodes (42.22%). Among them, single site metastasis accounted for 41.11% and multiple site metastasis 58.89%. 89 cases survived and 65 cases died. The survival time was 22 months to 60 months, and the median survival time was 48.0 months. The Breslow thickness, stage at diagnosis, distant metastasis, site of metastasis and ulceration were compared between the survival group and the death group (*P* < .05). serum lactate dehydrogenase (LDH), neutrophil-to-lymphocyte ratio (NLR) and lymphocyte monocyte ratio (LMR) were compared between the survival group and the death group (*P* < .05). The results of Logistic regression model showed that LDH ≥ 281 U/L, NLR ≥ 2.96, LMR ≤ 3.57, newly diagnosed stage > stage II, distant metastasis, multiple site metastasis and tumor ulcer were independent risk factors for poor prognosis of AM patients (*P* < .05). Patients with AM had a higher proportion of distant metastasis, mainly lung and lymph node metastasis. Increased LDH, increased NLR, decreased LMR, higher initial stage, distant metastasis, multiple site metastasis, and combined tumor ulcer were closely related to the poor prognosis of patients after surgery.

## 1. Introduction

Acral melanoma (AM) is a special subtype of melanoma, accounting for only 2% to 3% of melanoma.^[1,2]^ Unlike other types of melanoma that mainly occur in skin exposed to sunlight, AM mainly occurs on skin with less sunlight exposure, such as palms, soles of feet, fingers, toes, and nail units, most commonly in the lower extremities.^[3,4]^ The average age of diagnosis of AM is about 62.8 years old. The incidence of AM in all populations increases with age, and the incidence of AM in people over 80 years old increases sharply. And women are more likely to be diagnosed at an early stage than men.^[5]^ The main treatment of AM is surgical resection. According to the stage and characteristics of the tumor, chemotherapy, targeted therapy or immunotherapy can be used.^[6,7]^ Although great progress has been made in the treatment of melanoma in recent years, AM patients have shown limited benefits from current treatment, so the overall survival rate is poor.

Compared with other types of melanoma, the prognosis of AM is worse, mainly due to its late diagnosis and more invasive growth pattern.^[8,9]^ And its clinical features and prognosis are significantly different between different races. In fact, the US Surveillance, Epidemiology and End Results data report^[10]^ that clinical features such as AM pathological type, anatomical origin and patient outcome are significantly different between races. Due to genetic and environmental factors, including ultraviolet exposure and geographical location, acral and mucinous melanoma accounts for the majority of cases in the Asian population, which is characterized by late detection and poor prognosis.^[11]^ AM usually occurs in areas with little or no sunlight, accounting for about 41.8% of China’s cases.^[12]^ In western countries, superficial diffuse melanoma accounts for 50% to 70% of cases, while in East Asia, it accounts for only 5% to 37%. East Asian countries (including China, Japan, South Korea, and Singapore) report a lower incidence of melanoma (≤1.5/100,000).^[13,14]^ Due to its low incidence, data on the characteristics and clinical outcomes of AM are still limited in China. Therefore, this study aims to provide relevant theoretical support for the clinicopathological characteristics and prognosis of AM patients in China.

## 2. Data and methods

### 2.1. General data

In this study, 154 patients with AM admitted to hospital from January 2018 to April 2021 were selected for clinical study by retrospective study. This study was supported by the Ethics Committee of Nanjing Pukou People’s Hospital.

Inclusion criteria: (1) The diagnostic criteria of AM patients admitted to this study refer to the criteria in the “Melanoma Diagnosis and Treatment Specification 2018 Edition.”^[15]^ (2) Tumor lesions were located at the end of the limb (fingers, palms, toes, soles, etc). (3) All patients were treated with surgical resection in our hospital. The resection margin was ≥ 2.0 cm. Pathological examination was performed after resection to confirm the condition. (4) The age of patients ranged from 19 to 79 years old. (5) The study protocol was reviewed and approved by the Medical Ethics Committee.

Exclusion criteria: (1) Patients with other malignant tumors. (2) Major diseases associated with other systems (liver and kidney function diseases, blood system diseases, severe heart failure, etc). (3) Patients with immune system diseases. (4) Patients who failed to obtain follow-up data.

### 2.2. Surgical methods

*Surgical methods*: Before surgery, patients should undergo a thorough preoperative evaluation, including physical examination, imaging examination, and sometimes biopsy to determine the diagnosis and determine the characteristics of melanoma. The patient is placed under general anesthesia to ensure that the patient is unconscious and painless during the operation. An incision is made around the tumor. The size and shape of the incision depends on the characteristics of the tumor to achieve a clear edge. The tumor was carefully separated from the surrounding healthy tissue, and the edge of the tumor and the surrounding normal tissue was determined and removed according to the thickness of the tumor. Sentinel lymph node biopsy was performed at the same time as the extensive resection of the tumor. If the sentinel lymph node biopsy showed the presence of cancer cells, more extensive lymph node dissection was performed. During the operation, attention should be paid to control bleeding. After tumor resection, the wound at the resection site was evaluated and the corresponding reconstruction was performed.

### 2.3. Laboratory index detection methods

Before each outpatient follow-up, the patient was asked to be fasting, and 2 mL of fasting blood was collected and centrifuged at 2000 r/min for 10 minutes. The serum was immediately stored in a refrigerator at −80 °C. The levels of lactate dehydrogenase (LDH) were detected by Olympus AU5400 automatic biochemical analyzer, and the levels of ESR, neutrophil-to-lymphocyte ratio (NLR), lymphocyte monocyte ratio (LMR), and PLT were detected by Baden 5160 CS automatic blood cell analyzer. The test results were recorded in detail in the patient follow-up information.

### 2.4. Follow-up method

A total of 154 patients with AM were enrolled in this study. The follow-up time was ≥ 60 months, and the last follow-up time, death or loss of follow-up time was the end point of follow-up. A total of 154 patients were followed up, and all patients completed the follow-up. According to the research content, the laboratory examination and imaging examination data were improved during the follow-up process, and the clinical symptoms of the patients were recorded. This study was set as the main time node of follow-up from the time of enrollment to the time of distant metastasis and death of the patient. During the follow-up period, those who died of other causes than the disease itself, those who were lost to follow-up, or those who survived until the end of follow-up were marked as truncated values.

### 2.5. Statistical processing

The data were processed by SPSS21.0. The count data of the age index of the patients collected in this study were in accordance with the normal distribution, and the statistical description was completed by (‾χ ± s). The enumeration data (gender, Breslow thickness, initial diagnosis stage, distant metastasis, metastasis site, tumor ulcer) were described by the number of cases (percentage), and the χ2 test was used for comparison between groups. Logistic regression model was used to analyze the multivariate factors affecting the survival of AM patients.

## 3. Results

### 3.1. Distant metastasis of 154 AM patients

In this study, 154 patients with AM were treated, including 88 males and 76 females, aged from 27 to 79 years old, with an average age of (59.3 ± 11.7) years old. Among them, 90 cases had distant metastasis. The main metastatic sites were lung (47.78%) and lymph nodes (42.22%). Among them, 37 cases (41.11%) had single site metastasis, and 53 cases (58.89%) had multiple site metastasis. See Table 1.

**Table 1:** Distant metastasis of 154 AM patients.

Index	Cases	Proportion (%)
*Distant metastasis*		
Yes	90	58.44%
No	64	41.56%
*Transfer site*		
Lungs	43	27.92%
Liver	23	14.94%
Bone	19	12.34%
Lymph node	38	24.68%
Central nervous system	7	4.55%
Other parts	13	8.44%
*Number of transferred parts*		
Individual parts	37	41.11%
Multiple parts	53	58.89%

### 3.2. The 5-year survival of 154 AM patients

**Figure 1.**
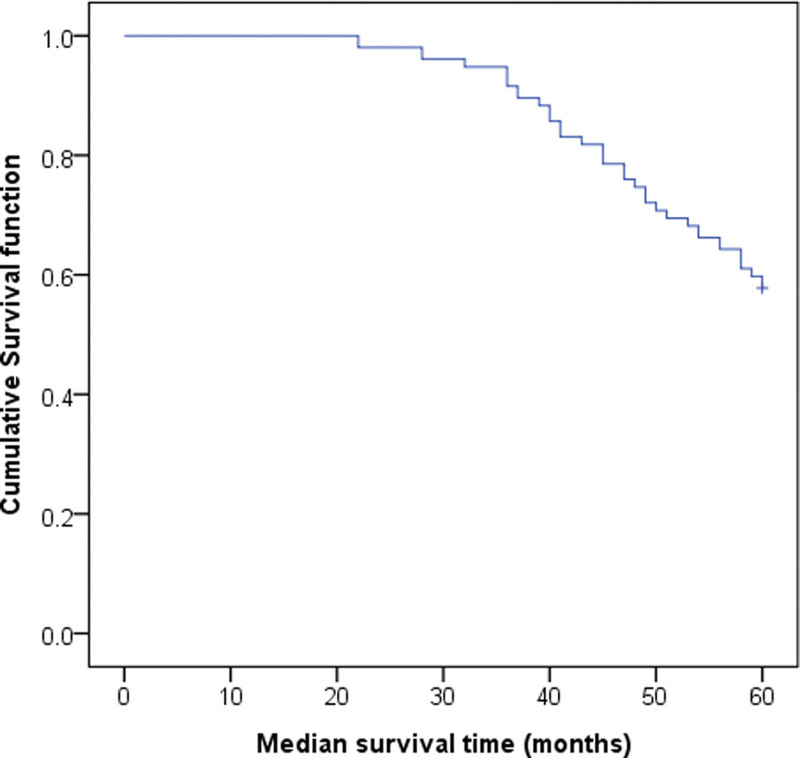
Survival function of 154 AM patients.

After follow-up, 154 patients with AM were followed up for 5 years, 89 patients survived and 65 patients died. The survival time was 22 months to 60 months, and the median survival time was 48.0 months. See Figure 1.

### 3.3. The general data and pathological differences affecting the survival of AM patients

The general data and pathological data of the surviving patients and the dead patients were compared. The age, sex, tumor location and lesion length of the 2 groups were compared (*P* > .05). The Breslow thickness, stage at diagnosis, distant metastasis, site of metastasis and ulceration were compared between the survival group and the death group (*P* < .05). See Table 2.

**Table 2:** The general data and pathological differences affecting the survival of AM patients [n(%)].

Index	Survival (n = 89)	Death (n = 65)	*χ* ^2^	*P*
*Age (years*)			2.472	.116
≥60	42 (47.19)	39 (60.00)		
<60	47 (52.81)	26 (40.00)		
*Gender*			2.564	.109
Male	46 (51.69)	42 (64.62)		
Female	43 (48.31)	23 (35.38)		
*Tumor site*			1.198	.274
Upper limb	35 (39.33)	20 (30.77)		
Lower limbs	54 (60.67)	45 (69.23)		
*Breslow thickness*			4.848	.028*
>T2	43 (48.31)	43 (66.15)		
≤T2	46 (51.69)	22 (33.85)		
*Initial diagnosis staging*			7.765	.005*
>II stage	43 (48.31)	46 (70.77)		
≤II stage	46 (51.69)	19 (29.23)		
*Distant metastasis*			5.739	.017*
Yes	43 (48.31)	44 (67.69)		
No	46 (51.69)	21 (32.31)		
Transfer site			5.367	.021*
≤1site	48 (53.93)	47 (72.31)		
Multiple sites	41 (46.07)	18 (27.69)		
*Tumor ulcer*			11.211	.001*
Yes	48 (53.93)	52 (80.00)		
No	41 (46.07)	13 (20.00)		
*Lesion length and diameter*			2.914	.088
>2.0 cm	54 (60.67)	48 (73.85)		
≤2.0 cm	35 (39.33)	17 (26.15)		

* *P* < .05.

### 3.4. Comparison of peripheral blood laboratory indexes affecting the survival of AM patients

The peripheral blood laboratory data of survival patients and death patients were compared, and the ESR and PLT values of the 2 groups were compared (*P* > .05). Serum LDH, NLR and LMR were compared between the survival group and the death group (*P* < .05). See Table 3.

**Table 3:** Comparison of peripheral blood laboratory indexes affecting the survival of AM patients[(%)].

Index	Survival (n = 89)	Death (n = 65)	*χ* ^2^	*P*
*ESR* (mm/h)			3.043	.081
≥30	51 (57.30)	28 (43.08)		
<30	38 (42.70)	37 (56.92)		
*LDH* (U/L)			9.935	.002*
≥281	36 (40.45)	43 (66.15)		
<281	53 (59.55)	22 (33.85)		
*NLR*			4.892	.027*
≥2.96	32 (35.96)	35 (53.85)		
<2.96	57 (64.04)	30 (46.15)		
*LMR*			4.703	.030*
>3.57	58 (65.17)	31 (47.69)		
≤3.57	31 (34.83)	34 (52.31)		
*PLT* (×10^9^/L)			3.380	.066
>257	32 (35.96)	33 (50.77)		
≤257	57 (64.04)	32 (49.23)		

* *P* < .05.

### 3.5. Multivariate analysis of factors affecting the survival of AM patients

Statistically significant serum LDH (<281 U/L = 0, ≥281 U/L = 1), NLR (<2.96 = 0, ≥2.96 = 1), LMR (>3.57 = 0, ≤3.57 = 1), Breslow thickness (≤T2 = 0, >T2 = 1), initial diagnosis stage (≤II = 0, >II = 1), distant metastasis (no = 0, is = 1), metastatic site (≤1 = 0, multiple = 1), tumor ulcer (no = 0, is = 1) were used as independent variables. Logistic regression model was established with 5-year follow-up outcome (survival = 0, death = 1) as the dependent variable. The results showed that LDH ≥ 281 U/L, NLR ≥ 2.96, LMR ≤ 3.57, initial stage > stage II, distant metastasis, multiple site metastasis and tumor ulcer were independent risk factors for poor prognosis of AM patients (*P* < .05). See Table 4.

**Table 4:** Multivariate analysis of factors affecting the survival of AM patients.

Factor	β	SE	Walds	*P*	OR	95% CI	
Breslow thickness	0.481	0.283	2.889	.097	1.618	0.929	2.817
Initial diagnosis staging	0.628	0.243	6.679	.000*	1.874	1.164	3.017
Distant metastasis	0.496	0.227	4.774	.038*	1.642	1.052	2.562
Transfer site	0.443	0.195	5.161	.031*	1.557	1.063	2.282
Tumor ulcer	0.581	0.243	5.717	.008*	1.788	1.110	2.879
LDH	0.633	0.26	5.927	.004*	1.883	1.131	3.135
NLR	0.492	0.231	4.536	.044*	1.636	1.040	2.572
LMR	0.771	0.301	6.561	.000*	2.162	1.198	3.900

* *P* < .05.

## 4. Discussion

AM is generally considered to be more invasive. Although the metastasis rate of AM is lower than that of other types of melanoma, it can still metastasize in some cases.^[16,17]^ The most common metastatic sites of AM include regional lymph nodes, lungs, liver and brain. However, the specific pattern and prevalence of transfer may vary from person to person.^[18,19]^ This study found that patients with AM are prone to distant metastasis, which is mainly located in the lungs (47.78%) and lymph nodes (42.22%), and mainly multi-site metastasis (58.89%). The results of this study are basically consistent with the previous findings.^[20]^ On the one hand, this is related to the stronger invasiveness of AM. On the other hand, according to the characteristics of blood circulation and lymphatic reflux, it is more likely to have lung and regional lymph node metastasis first. This distant metastasis characteristic of AM is often one of the important factors for poor prognosis of patients.^[21]^

The prognosis of AM also depends on a variety of other factors, including region, race, gender, age, etc,^[1]^ as well as the stage of cancer at the time of diagnosis,^[1]^ Breslow thickness,^[22]^ combined ulcers,^[23]^ pathological stage,^[24]^ and sentinel lymph node positive^[25]^ and the overall health status of the individual. Studies have shown that,^[26]^ sentinel lymph node positive is the strongest predictor of melanoma disease recurrence and death.^[27]^ AM usually appears in the later stage, which is also considered to be one of the reasons for its poor prognosis. The results of this study showed that tumor Breslow thickness > T2, late stage of initial diagnosis, multiple distant metastasis, and the presence of tumor ulcers were important factors for the poor prognosis of AM patients, and were positively correlated with increased mortality, while the patient’s age, gender, tumor location, and size were not significantly correlated with it. Therefore, compared with demographic factors, the prognosis of AM is more correlated with its own biological characteristics. Due to the stronger invasion ability of tumor cells, it means easier and faster metastasis and stronger vitality of distant metastases. Therefore, under the same premise, AM is more common with increased Breslow thickness, advanced diagnosis, multiple distant metastases, and presence of tumor ulcers, and ultimately leads to high mortality.

LDH, NLR and LMR are associated with poor prognosis and poor survival outcomes in a variety of cancers, including lung cancer, colorectal cancer, melanoma, lymphoma, etc, and are considered as potential prognostic markers for a variety of cancers.^[28–31]^ LDH is an enzyme involved in the conversion of lactic acid to pyruvate and plays a crucial role in cellular energy metabolism.^[32]^ It is also a marker of tissue damage and cell death, and elevated levels of cell death may reflect increased tumor burden, hypoxia, and metabolic activity.^[33]^ NLR is considered to be a marker of systemic inflammation and immune response, and its elevated level indicates an imbalance between systemic inflammation and the pro-tumor effect of neutrophils and the antitumor effect of lymphocytes.^[34]^ LMR is considered to be a marker of the balance between lymphocytes and monocytes. Lymphocytes are important in anticancer immune responses, while monocytes may have tumor-promoting properties.^[35]^ In this study, univariate and multivariate analysis showed that serum LDH, NLR, LMR, initial stage, distant metastasis, multiple metastasis and combined tumor ulcer were independent factors related to the prognosis of AM. Studies have shown^[36]^ that higher LDH levels in AM patients are associated with advanced disease, thicker tumor depth, ulcers, lymph node involvement, and distant metastasis. Increased LDH levels may indicate that AM has more aggressive tumor behavior and worse prognosis. The increased level of NLR indicates the imbalance of immune response and the increase of systemic inflammation, which contributes to the progression and metastasis of tumors. The decreased LMR level indicates insufficient immune response and high systemic inflammation, which is not conducive to controlling tumor growth and progression. Therefore, the AM patients with poor prognosis are more malignant and invasive, and their autoimmune mobilization is worse, and the systemic inflammatory response is more obvious. In addition, the independent risk factors are interrelated, but their specific relevance still needs more in-depth study.

In summary, the proportion of distant metastasis in AM patients is higher, and it is mainly lung and lymph node metastasis. The increase of LDH, the increase of NLR, the decrease of LMR, the higher the initial stage, the occurrence of distant metastasis, the occurrence of multi-site metastasis, and the combination of tumor ulcers are closely related to the poor prognosis of patients after surgery. However, our research still needs to be improved, and more data are needed to support this result.

## Author contributions

**Conceptualization:** Yan Fang, Wu Di.

**Data curation:** Zhu Yongqian.

**Formal analysis:** Lu Yin.

**Investigation:** Min Li.

**Supervision:** Zhang Mei, Yang Jing.

**Writing – original draft:** Yan Fang.

**Writing – review & editing:** Yan Fang, Wu Di.
